# Novel Nanotechnology-Based Approaches for Targeting HIV Reservoirs

**DOI:** 10.3390/polym14153090

**Published:** 2022-07-29

**Authors:** Leila Fotooh Abadi, Fouad Damiri, Mehrukh Zehravi, Rohit Joshi, Rohan Pai, Mohammed Berrada, Ehab El Sayed Massoud, Md. Habibur Rahman, Satish Rojekar, Simona Cavalu

**Affiliations:** 1 Department of Virology, Indian Council of Medical Research, National AIDS Research Institute, Pune 411026, Maharashtra, India; elmiramil@gmail.com; 2 Laboratory of Biomolecules and Organic Synthesis (BIOSYNTHO), Department of Chemistry, Faculty of Sciences Ben M’Sick, University Hassan II of Casablanca, Casablanca 20000, Morocco; berrada_moh@hotmail.com; 3 Department of Clinical Pharmacy Girls Section, Prince Sattam Bin Abdul Aziz University, Alkharj 11942, Saudi Arabia; mahrukh.zehravi@hotmail.com; 4 Precision NanoSystem Inc., Vancouver, BC V6P 6T7, Canada; rohitjoshi07@gmail.com; 5 Shobhaben Pratapbhai Patel School of Pharmacy & Technology Management, SVKM’s NMIMS, V.L. Mehta Road, Vile Parle (W), Mumbai 400056, Maharashtra, India; rohanvpai@gmail.com; 6 Biology Department, Faculty of Science and Arts in Dahran Aljnoub, King Khalid University, Abha 62529, Saudi Arabia; ehabma@kku.edu.sa; 7 Research Center for Advanced Materials Science (RCAMS), King Khalid University, Abha 61413, Saudi Arabia; 8 Agriculture Research Centre, Soil, Water and Environment Research Institute, Giza 3725004, Egypt; 9 Department of Global Medical Science, Wonju College of Medicine, Yonsei University, Gangwon-do, Wonju 26426, Korea; pharmacisthabib@gmail.com; 10 Department of Pharmaceutical Sciences and Technology, Institute of Chemical Technology, Mumbai 400019, Maharashtra, India; 11 Departments of Medicine and Pharmacological Sciences, Icahn School of Medicine at Mount Sinai, New York, NY 10029, USA; 12 Faculty of Medicine and Pharmacy, University of Oradea, P-ta 1 Decembrie 10, 410087 Oradea, Romania

**Keywords:** active targeting, HIV reservoirs, nanomedicine, passive targeting, phagocytosis, viral infection

## Abstract

Highly active anti-retroviral therapy (HAART) is prescribed for HIV infection and, to a certain extent, limits the infection’s spread. However, it cannot completely eradicate the latent virus in remote and cellular reservoir areas, and due to the complex nature of the infection, the total eradication of HIV is difficult to achieve. Furthermore, monotherapy and multiple therapies are not of much help. Hence, there is a dire need for novel drug delivery strategies that may improve efficacy, decrease side effects, reduce dosing frequency, and improve patient adherence to therapy. Such a novel strategy could help to target the reservoir sites and eradicate HIV from different biological sanctuaries. In the current review, we have described HIV pathogenesis, the mechanism of HIV replication, and different biological reservoir sites to better understand the underlying mechanisms of HIV spread. Further, the review deliberates on the challenges faced by the current conventional drug delivery systems and introduces some novel drug delivery strategies that have been explored to overcome conventional drug delivery limitations. In addition, the review also summarizes several nanotechnology-based approaches that are being explored to resolve the challenges of HIV treatment by the virtue of delivering a variety of anti-HIV agents, either as combination therapies or by actively targeting HIV reservoir sites.

## 1. Introduction

### 1.1. Human Immunodeficiency Virus (HIV)

HIV infection, commonly referred to as acquired immune deficiency syndrome (AIDS), is one of the utmost challenging diseases of the 21st century, with severe social, financial, and political implications in developed and developing nations [1,2,3,4,5,6,7]. As it is an immunological disorder, it weakens the immune system, thereby increasing the risk of death due to opportunistic comorbidities, such as tuberculosis, septicemia, and pneumonia [8].

**Figure 1:**
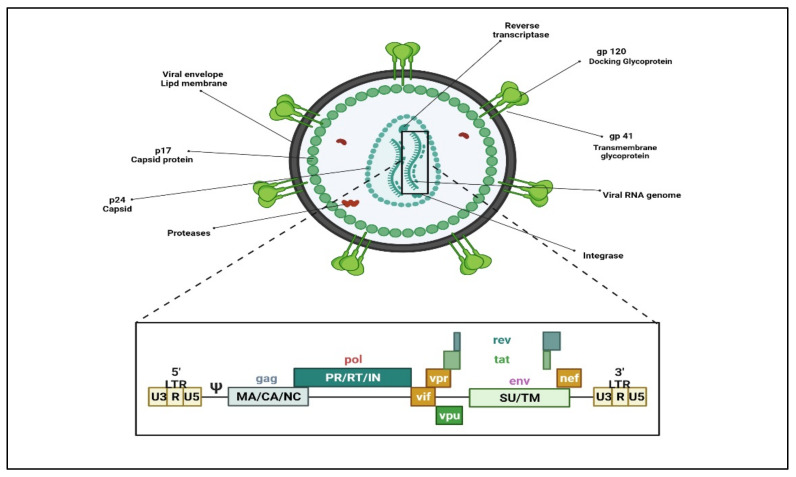
HIV virion structure and genome organization. (Created with BioRender.com (accessed on 19 June 2022)).

HIV (HIV-1 and HIV-2) belongs to the retroviruses family within the genus lentivirus. As with all retroviruses, the genome in the virus particle is diploid, comprising of two single-stranded RNA molecules. On infection, the viral enzyme reverse transcriptase catalyzes the synthesis of a haploid, double-stranded DNA provirus that becomes inserted into the chromosomal DNA of a host cell and tricks the host biosynthetic machinery for its protein synthesis. The genome contains 10 open reading frames, encoding several crucial viral gene products that provide structural integrity to the virion and enzymatic functions for viral replication or regulating viral gene expression. (Figure 1) Reverse transcriptase (RT), the viral polymerase responsible for retro-transcribing viral RNA to double-stranded DNA, lacks proofreading, thus making the process of viral genetic replication susceptible to errors. Viral subtypes are formed when such errors steadily accumulate, gradually leading to the evolution of viruses that could vary significantly from one another at the genetic level.

HIV is the only human virus that infected T-cell lymphocytes and spread sexually and via infected blood or from infected mother to fetus. Prior to being implicated as the causative agent of AIDS, the role of Epstein–Barr Virus (EBV), cytomegalovirus, and Hepatitis B Virus (HBV) were investigated for the etiology of AIDS. Scientists eventually noticed that T-helper lymphocytes were decreased in these patients, and AIDS was attributed to human T-lymphotropic viruses. Thus, it was hypothesized that the etiology of AIDS was a variant of HTLV-III and was explained as HIV [9,10]. Human HIV-2 infection and simian immunodeficiency virus in Macaque (SIVmac) result from the cross-species transmission. The evidence of cross-species transmission is provided by phylogenetic relationships between SIVcpz and SIVsm, which are of the same lineage as HIV-1 and HIV-2, respectively. Nucleotide sequencing information on the envelope (Env) protein of different HIV isolates showed so much variability that sub-classification of HIV-1 strains became necessary. Based on a nucleotide sequence, mainly of the *env* gene, HIV-1 could be subdivided into “subtypes” or “clades.”

There are three main groups within HIV-1, i.e., M (major), N, and O (outliner). M comprises most of the HIV-1 strains, which comprise at least 10 to 12 genetically separate sub-types labeled A through J. Additionally, group O consists of a separate group of viruses recognized primarily in Cameroon. A small number of strains genetically different from the above two groups have primarily been limited to Cameroon and Gabon. These are located in group N. Nevertheless, the divergence of sub-types is a dynamic, continuing process, and many viral sub-types are still being identified. Numerous strains persist uncategorized since they fail to segregate with any known groups.

### 1.2. Replication Cycle

**Figure 2:**
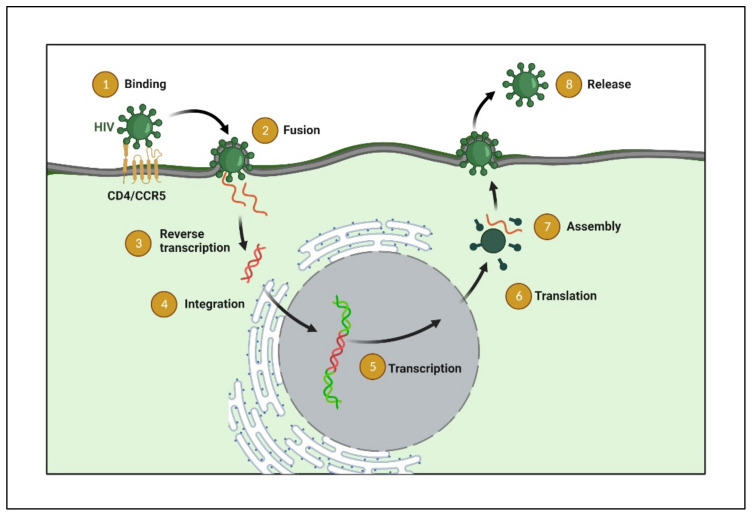
Infographic illustration of the HIV replication cycle begins with the fusion of HIV with the host cell surface, leading to the entry and release of the virus’s genome and proteins in the form of a capsid. The capsid shell disintegrates, and the HIV reverse transcriptase transcribes the viral RNA into DNA. The viral DNA is transported across the nucleus and integrated into the host’s DNA via the HIV protein integrase. It utilizes the host’s normal transcription machinery to transcribe the HIV DNA into multiple new HIV RNA copies. This RNA can be packaged as a new virus genome or utilized by the cell to make new HIV proteins. The new viral RNA and HIV proteins translocate to the cell surface to form a new, immature HIV virion. Finally, the HIV protease cleaves these newly translocated polyproteins to create a mature infectious virus released from the cell; different stages can be an intervention site for different ARTs. (Created with BioRender.com (accessed on 19 June 2022)).

There are six steps involved in the HIV lifecycle: binding and fusion, reverse transcription, integration, transcription, and translation, assembly, and budding, as shown in Figure 2 [11]. Briefly, the HIV life cycle begins with its entry into target cells initiated by HIV-1 gp120 binding to the cell surface receptors (CD4) and co-receptors (CCR5 and CXCR4). Following viral entry, replication and assembly are mediated by numerous enzymes, such as reverse transcriptase, protease integrase, and ribonuclease. The pre-integration complex docks the nuclear membrane and enters the nucleus through the nuclear pore. Viral integrase inserts linear double-stranded DNA present in the pre-integration complex in the host chromosome. 

Upon activating the host cell, the virus uses host enzymes and produces more of its genetic material and longer HIV proteins. Proteases further cut the longer HIV proteins in the smaller individual proteins. Lastly, these proteins with the virus’s genetic material assemble into the new virus. Several enzymes involved in the HIV life cycle present validated targets for different ART, which may interfere at one or more stages of the HIV life cycle as per their mode of action (Figure 2) and are further touched upon in this review.

### 1.3. Epidemiology of HIV/AIDS

As per the World Health Organization (WHO), there were 37.7 million HIV-infected people at the end of 2021. Approximately 0.8% of adults aged 15–49 years are living with HIV, and more than half (53%) are women [12,13]. Sub-Saharan Africa continues to lead the statistics accounting for 71% of the afflicted population. The retrovirus diffused via sexual contact, needles (tattooing, body piercing, and sharing for drugs), blood transfusions, and from mother to fetus in infected individuals throughout pregnancy and breastfeeding [4,14,15].

### 1.4. HIV Niches

**Figure 3:**
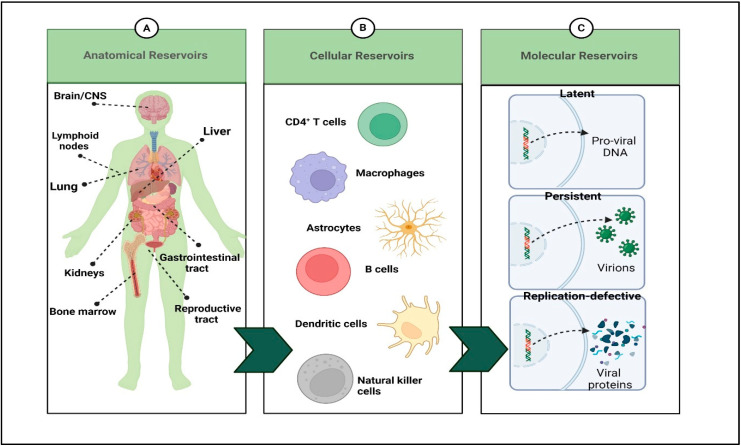
Summary HIV reservoirs. There are several anatomical compartments (**A**) that are populated by HIV-infected cells (**B**). (**C**) The integrated provirus contained within these cells may be transcriptionally silent (latent), transcriptionally active and capable of producing infectious virions (persistent), or transcriptionally active but replication defective due to mutations or deletions in the HIV genome, leading to translation of specific viral proteins for which an open reading frame remains intact. (Created with BioRender.com (access on 19 June 2022)).

Unlike most other infections, a typical feature of HIV is propagation across the body into various anatomical sites and different cell types. Moreover, these locations protect the virus from therapeutic challenges by limiting drug access, thus enabling their rapid multiplication and increasing the severity of the infection. Several sites could serve as reservoirs of HIV replication, comprising tissues (i.e., Lymph nodes, liver, lungs, etc.) and cells (i.e., CD4^+^ cells and Astrocytes, etc.). However, HIV invasion includes remote sites, i.e., the brain/central nervous system (CNS), reproductive tract, and bone marrow (Figure 3) [1,16,17]. 

These sites are immunologically shielded or isolated by a barrier from blood and lymphoid systems and the phrase “HIV reservoir” has widely been exploited to designate the cells and tissues that endure retaining HIV after receiving optimal treatment [14].

#### 1.4.1. Anatomical Reservoirs

##### Lymphoid Tissues

HIV usually gains entry into the human host through mucosal surfaces, and its subsequent dissemination all through the lymphatic tissues forms a major reservoir during infection [15,16]. The thymus and bone marrow constitute primary or central lymphoid organs. In contrast, lymph nodes and lymphoid follicles in the spleen, skin, Peyer’s patches, and adenoids are mucosa-associated lymphoid tissues that form secondary or peripheral lymphoid organs [17,18]. Along with being the primary viral replication site, these tissues also contain the bulk of infected cells and free virions captured via the follicular dendritic cell network. Dendritic cells and macrophages facilitate the colonization of the virus in lymphoid tissue, further passing the virus to the lymph nodes where initial CD4^+^ T lymphocytes infection occurs as about 98% CD4^+^ population resides in lymphoid tissue [19]. In the case of bone marrow, HIV can also hide inside hematopoietic progenitor cells (HPCs), presenting another challenge in HIV eradication [20]. The monocyte-macrophage lineage cells in the bone marrow also play a vital role in spreading the infection [21], while conflicting results about the involvement of mast cells have been reported [22] Lower ART drug levels were observed in comparative studies in lymph nodes and the spleen of humans and SIVmac highlight the impermeability of the secondary lymphoid tissue to ART [23,24].

##### Liver

The liver is critical in the pathogenesis of HIV infection. However, a detailed evaluation of HIV replication in liver tissue is absent, leading to several possibilities. The liver contains T cells, macrophages (i.e., Kupffer cells), and other immune cells, which have higher infection rates than hepatocytes [25]. The liver empties most of the intestine blood and is the leading site of CD4^+^T lymphocytes reduction and increase in the virus in the initial stage of infection. The T cells’ normal composition in the liver is altered markedly in early infection, possibly due to opportunistic infections [26]. 

##### Gastrointestinal Tract

The gut is another area of interest as it contains up to 90% of lymphoid tissue and associated cells and is estimated to carry around 90% of all the HIV-infected CD4^+^ T cells [19]. Viruses readily target CXCR4 and CCR5 target cells prevalent in the gastrointestinal mucosa, enabling translocation across the epithelium [27,28].

##### Lung

Alveolar macrophages and alveolar lymphocytes form sanctuary sites in the lung for HIV [29,30].

##### Kidneys

The kidney denotes another site of HIV replication and remains and commands particular interest because of the HIV-associated nephropathy. Renal tubular epithelial and glomerular cells have been reported to harbor HIV DNA and HIV mRNA, indicating prolific infection [31].

##### Central Nervous System (CNS)

The CNS is a crucial anatomical reservoir site, where microglia and macrophages are major brain reservoirs for HIV infection and replication. HIV invades the CNS through peripherally infected leukocytes, mainly monocytes. AIDS-related dementia complex, a poorly understood late-stage HIV complication, is known to manifest itself over several months [32,33]. 

##### Reproductive Tract

Male and female genital tracts are latent reservoirs for HIV [34,35]. A lack of relationship between plasma and semen viral loads suggested independent viral replication. CD4-receptors present on the surface of lymphocytes and macrophages infiltrate the testes. HIV infection in the female genital tract has been demonstrated in various tissues, including stromal cells of the uterus, epithelial cells, cervix, fallopian tube, and ectocervix [36,37].

#### 1.4.2. Cellular Reservoirs

A wide range of cell types distributed in several tissues is vulnerable to HIV infection. Macrophages and CD4^+^ T lymphocytes are the principal cellular targets of HIV, followed by natural killer (NK) cells, CD8^+^ T lymphocytes, monocytes, peripheral blood, follicular-dendritic cells, and B cells. Additionally, an array of specific cell types is derived from various tissue reservoirs of HIV (i.e., mucosal, cervical epithelial and renal cells, astrocytes and microglia in the CNS, bone marrow stem cells, and skin fibroblasts) are also reported to harbor the virus [38,39,40,41] (Figure 3B).

##### CD4^+^ T Lymphocytes

The symbol of HIV infection is the continuous reduction in CD4^+^ T lymphocytes. CD4^+^ T cells residing in lymph nodes, gastrointestinal mucosa, and intestine are vital sites of HIV replication in vivo [42,43], and memory CD4^+^ T cells and macrophage–monocyte cells act as latent virus reservoirs. [44,45].

##### Monocytes and Macrophage Lineage

Monocyte macrophage lineage forms a critical HIV reservoir. Monocytes circulate in the blood for 3 days and are differentiated into macrophages upon migration into various tissues. Macrophages colonize in the liver (kupffer cells), spleen (sinusoidal lining cells), lungs (alveolar macrophages), central nervous system (microglia), lymph nodes (sinus histiocytes), blood (monocytes), placenta (hofbauer cells) osteoclasts in bone [46], and testicular macrophages. They can act as latent vaccine reservoirs [44,45].

##### Dendritic Cells

Dendritic Cells (DCs) are antigen-presenting cells that show a significant role in HIV pathogenesis. A DCs subset of Langerhans cells, located in epithelial surfaces, such as skin, present an initial target for HIV infection. Myeloid DCs, plasmacytoid DCs, and antigen-presenting cells form additional targets of HIV. DCs capture virions in the mucosa during a normal immune response and migrate to the lymphoid tissue. In the lymphoid tissue, DCs infects CD4^+^ T lymphocytes on a large scale. Several receptors facilitate viral attachment on the surface of DCs, viz., C-type lectins, comprising DC-SIGN (dendritic cell-specific intercellular adhesion molecule-3-grabbingnonintegrin), d-mannose, and langerin [47,48].

##### B Lymphocytes Cells

B cells in lymphoid tissue have peripheral blood transport viruses attached to their surfaces [49].

##### Natural Killer (NK) Cells

NK cells are phenotypically defined as lymphocytes expressing the antigens CD56 and mostly CD16 (Fc gamma RIII) but lacking CD3 (CD3^−^ CD16^−^ CD56^+^). NK cells are expressing both CD4 and chemokine co-receptors CCR5 and CXCR4 demonstrated the presence of viral DNA [50].

#### 1.4.3. Molecular Reservoirs

These reservoirs are classified into latent, persistent, and replication-defective types at the molecular level. The virus acquires full transcriptional silencing in a latent reservoir and must be triggered to unleash a replicating viral particle. This concept is accomplished through the HIV infection of resting memory CD4^+^ T cells. Any long-lived cell infected with the virus, in contrast, could act as a chronic viral reservoir, releasing tiny quantities of virus consistently or sporadically [51] (Figure 3C).

### 1.5. Tackling the HIV: Challenges

**Figure 4:**
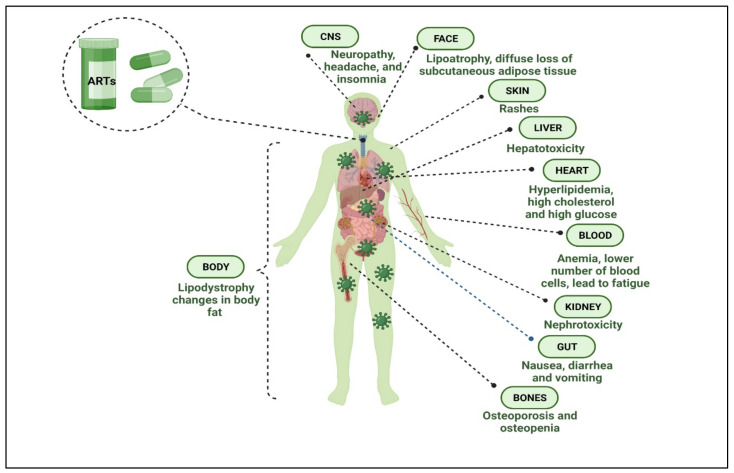
The illustration shows the major side effects of antiretroviral drugs on different body sites in an HIV/AIDS individual. (Created with BioRender.com (access on 19 June 2022)).

The current state of the art therapy for HIV/AIDS is HAART, which includes a combination of three to five agents targeting different viral proteins. It has significantly decreased HIV and associated disease morbidity and mortality rates [52,53,54]. This therapy has several limitations, such as toxicity, development of drug resistance, and poor tolerability [55]. Antiretroviral drugs include several classes, such as nucleoside reverse transcriptase inhibitors (NRTIs), non-nucleoside reverse transcriptase inhibitors (NNRTIs), protease inhibitors (PIs), and fusion inhibitors (FIs), co-receptor inhibitors (CRIs) [56]. The current HAART, based on drugs and drug combinations, as shown in Table 1, is challenged by high failure rates typically due to poor patient compliance and resistance [57]. High doses and resulting systemic toxicities coupled with the high cost of therapy are other significant issues. Patients on HAART have been reported to have higher rates of heart disease, neurological complications, diabetes, liver illness, cancer, and premature aging [51,52,53]. Figure 4 summarizes the side effects associated with HAART in HIV/AIDS individuals.

The specialists generally agree that most of these side effects could be triggered by HIV infection or co-infection with other viruses, such as co-infection with hepatitis C virus, which promotes liver damage. Furthermore, via encouraging inflammation and producing neurotoxins, monocyte and macrophage infection is implicated in HIV pathologies, comprising the progress of HIV-associated dementia (HAD) [58,59]. Conversely, the toxicity of the drugs employed in HAART could play a role in these side effects. The complete elimination of the virus from the body is not possible with current therapies due to the latency of the virus. Latent HIV-infected cells are often located in the secondary lymphoid tissue, testes, liver, kidney, lungs, gut, and central nervous system (CNS) [60,61,62,63,64,65]. The elimination of the virus from such reservoirs is crucial for a successful long-term treatment. Hence, innovative initiatives for generating nontoxic, low-dose therapy modalities that provide better-sustained dosage distribution and reliably eliminate the virus from reservoirs without requiring lifelong treatments are the need of the hour [66,67,68].

The main challenges regarding the consumption of HAART can be summarized as follows.

#### 1.5.1. Low Oral Bioavailability

Despite the remarkable advances in HIV therapy, ART implementation faces various hurdles. Although the oral ART dose is easy to consume, the administered drug undertakes significant first-pass metabolism over the oral route. ART inhibitors generally reach peak blood/plasma concentration (Tmax) in under an hour; however, their bioavailability varies widely (60–90%) subject to the absorption region [57,69,70]. Multidrug-resistant efflux proteins (MRPs) on the gastrointestinal tract, such as P-glycoprotein (P-gp), affect their oral bioavailability and concentration in CNS. The strong affinity towards protein seen in most anti-HIV drugs prevents their diffusion through the Blood–Brain Barrier (BBB), further decreasing its bioavailability [71,72]. Another severe factor with anti-HIV medication therapy is resistance. HIV replication is a fast and error-prone process (every day, 10 billion virus particles are generated), resulting in at least one mutation per genome. These genetic alterations allow the virus to acquire resistance to antiretroviral medication, particularly when monotherapy is used [69,70,73]. Drug resistance is becoming more widespread, and recently infected patients are at risk of acquiring an HIV-resistant strain [74,75].

#### 1.5.2. Long-Term Drug Therapy

Although AIDS-related morbidity and mortality have dropped considerably because of antiretroviral medication, it is vital to provide continuous treatment and combine drugs from several classes to avoid resistance generation [76,77]. Furthermore, long-term use of ARTs in people living with HIV (PLWH) are more likely to develop comorbidity related to aging, metabolic problems, chronic HIV, and the toxicity of long-term ARTs. Additionally, PLWH is now undergoing ART for considerably extended periods, and the possible cumulative toxicity that may follow is still uncertain [78,79]. On the other hand, compliance difficulties become a complication and are typically problematic [80,81,82]. Moreover, antiretroviral drugs have no impact on the virus living within macrophages. These viral reservoirs persevere after that initial anti-retroviral treatment onset, making antiretroviral therapy incredibly difficult [83,84,85]. 

#### 1.5.3. Toxicity

HIV can cause liver damage and consequent liver fibrosis (LF) in various ways. In addition to HIV and viral hepatitis, alcoholic and particularly nonalcoholic liver illnesses were linked to liver disease in PLWH. Moreover, ART is a documented source of hepatotoxicity, which raises clinically significant distresses regarding LF in long-term treatment [86,87]. ART can have a wide range of adverse effects, ranging from mild intolerance that can be self-limited to life-threatening complications. It might be challenging to differentiate between HIV-related problems and ART toxicity (also known as adverse reactions). A concurrent infection (i.e., common pediatric infections, such as hepatitis A in a child with hepatitis signs or malaria in a child with severe anemia) or the reported toxicity could be explained by a reaction to drugs other than ARTs. The symptoms of adverse reactions that are not induced by an ART drug do not necessitate switching ART [78,88]. The most common toxicities include mainly mitochondrial toxicity, hypersensitivity, lipodystrophy, dyslipidemia, and type 2 diabetes, which have all been poorly explored, analyzed, and published so far. This is partly attributed to the uncommonly high long-term and uncommon adverse effects further complicated by comorbidities in HIV patients [89]. Lactic acidosis, liver toxicity, pancreatitis, and peripheral neuropathy are all symptoms of mitochondrial dysfunction caused by NRTI drugs [90,91,92]. Lipodystrophy and other metabolic abnormalities are most familiar with PI’s and to a lesser extent with other NRTIs, especially Stavudine (d4T). Certain fatty tissue anomalies appear to be associated with this older class of ARTs [93,94]. Fat misdistribution and changes in body habitus, hyperlipidemia, hyperglycemia, insulin resistance, and diabetes mellitus are indeed abnormalities. However, allergic responses, including skin rashes and hypersensitivity reactions, are another prevalent ART toxicity, commonly seen with NNRTIs [95], but can also arise with NRTIs, such as abacavir (ABC) [96,97,98].

### 1.6. Nanopharmaceuticals; Novel Directions on HIV/AIDS Treatment Approaches

**Figure 5:**
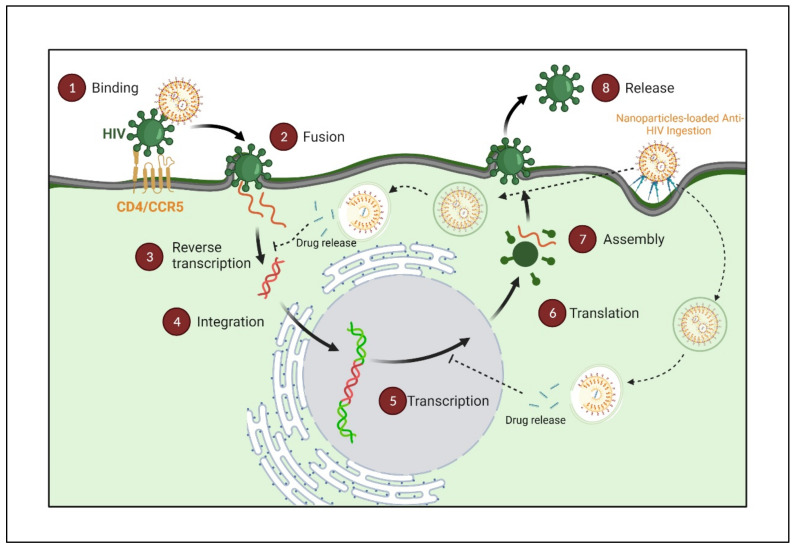
HIV sites for therapeutic intervention using nanopharmaceuticals. (Created with Biorender.com (access on 19 June 2022)).

Novel ART approaches and nanopharmaceuticals have displayed promising results to a certain extent in HIV/AIDS therapy. Nanomedicine is a field of medicine that employs nanotechnology for the prevention and treatment of diseases utilizing NPs, such as biocompatible NPs [99] and nanorobots [100] for numerous applications comprising diagnosis [101], delivery [102], sensory [103], or the actuation purposes in a living organism [104]. Nanomedicine provides novel approaches to preventing viral infection, growth, and transmission. Many inorganic and metal NPs (i.e., gold, silver, and silica nanoparticles) have been intensively studied for application in imaging, bioassays, and therapeutics [105,106,107,108,109]. Despite significant advances in HIV/AIDS treatment, there are many lacunas in the HIV/AIDS treatments addressed by nanoart, which target different stages of the HIV lifecycle as per the drug/s mechanism of action [110] (Figure 5).

### 1.7. Nanoparticles Transport Approaches

**Figure 6:**
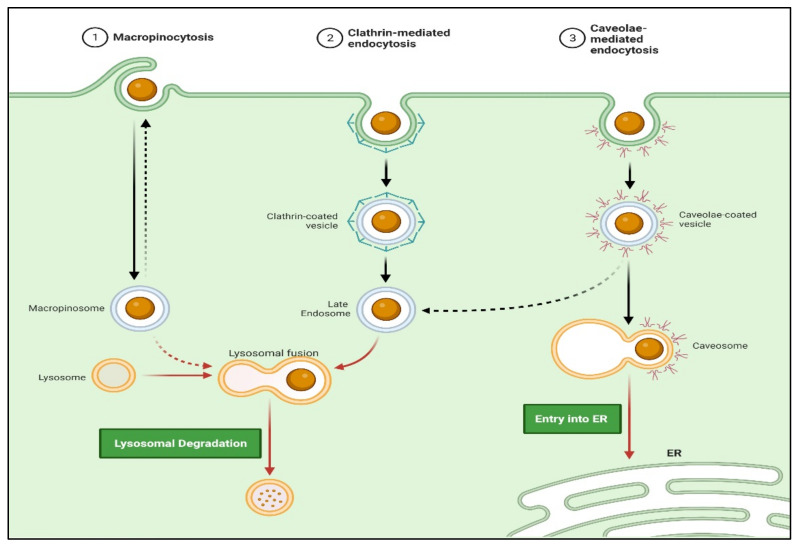
Active and passive uptake of nanoparticles to the HIV-infected tissues/organs. The active targeting strategy can include nanoparticle functionalization directly or indirectly with various molecules; such as drugs, nucleic acids (DNA or RNA), proteins or peptides, antibodies, etc., for ideal biological activities and diverse medical applications. (Created with BioRender.com (access on 19 June 2022)).

Most nanoparticulate drug delivery systems need to cross the cell membrane and deliver the cargo (ART) to elicit an antiviral response. Hence it is imperative to familiarize oneself with the nanoparticle transport approaches. There are multiple ways for nanoparticles to cross the cell membranes, primarily through paracellular or transcellular pathways (Figure 6). The majority, however, reach the target cells via a substrate-specific process known as the endogenous transporter or carrier-mediated route determined by the concentration gradient of the substrates with the help of appropriate transporters [96,111,112].

#### 1.7.1. Active Transport

Active targeting involves the specific modification of drug/drug-loaded nanocarriers with site-specific ligands or “active” agents, which have a discriminating or specific affinity for distinguishing and interacting with a specific cell, tissue, or organ in the body based on its protein expression profile [113]. An active targeting approach significantly increases the possibility of the drug target cell’s interaction while sparing healthy cells. The active targeting of particulate carriers can be further classified as;

##### Stimuli-Responsive Nanocarriers

Stimuli-sensitive nanocarriers are based on internal and external stimuli. The internal stimulus includes tumor microenvironment pH and temperature, while the external stimulus includes hyperthermia magnetic field or ultrasound energy [97,98,99,114,115,116].

##### Antibody Targeted Nanocarriers

An antibody having a specific affinity towards an antigen found on the target cell’s surface could be anchored on the nanocarriers’ surface to increase their targeting efficiency. Earlier, this approach was widely explored in cancer therapy, but currently, it has become a key interest in HIV/AIDS therapy. HIV-infected cells express various molecules, such as gp120, the HLA-DR determinant of MHC-II, and CD receptors on their surface, which may be targeted by whole antibodies or fragments of an antibody. Immunoliposomes are widely explored nanocarriers amongst various nanocarriers. Cells, such as follicular dendritic cells, B cells, and macrophages can express the HLA-DR determinant of MHC-II and be theoretically targeted by anti HLA-DR monoclonal antibodies. Immunoliposomes anchored with anti-HLA-DR monoclonal antibodies revealed enhanced accumulation of Indinavir in the lymph nodes, with greater than a 126-fold area-under-the-curve than the free drugs in mice. Kumar et al. demonstrated the effective suppression of HIV infection in humanized mice by targeted siRNA delivery to T cells using an antibody (scFvCD7) specific to the CD7 receptor on the T cells [100,101,102]. 

##### Receptor-Mediated Endocytosis (RME)

Target cells express various receptors on their surface to enable the internalization of drug-loaded cargoes into the cell and their degradation. The binding of ligand conjugated nanocarriers to receptors present on the cell surface generates a sequence of cellular actions that result in their internalization within the cell. Phagocytic processes are faster than RME, with the ligand playing an essential role in RME. The common RME mechanisms are micropinocytosis, clathrin-dependent endocytosis, caveolae-mediated endocytosis, and clathrin-independent endocytosis. Further, the uptake mechanism is often dependent on the nature of the ligand [96]. Macrophages are the primary differentiating cells of the mononuclear phagocyte system and are also responsible for disseminating the infection throughout the body, as mentioned elsewhere. Macrophages residing in the above-mentioned organs serve as a potential reservoir for HIV [97,98,113]. Targeting anti-HIV1 drugs to these macrophages residing in multiple HIV reservoirs would significantly benefit the therapy because many anti-HIV1 drugs administered via the conventional routes fail to penetrate these sites optimally. Table 2 summarizes some of the crucial receptors on macrophages and their respective ligands targeted for the treatment of infectious diseases. Targeted drug delivery may improve efficacy, drug resistance, reduction in dosage, and systemic toxicity and hence patient compliance [114,115,116]. Table 3 outlines the most important nanocarriers for anti-HIV1 drugs and their target sites.

##### The d-Mannose Receptor Targeting

The d-mannose receptor (MR, CD206, or MRC1) is a transmembrane glycoprotein classified under the C-type lectin family and present on most macrophages’ surfaces. Its extracellular regions consist of an N-terminal cysteine-rich (CR) domain, which has an affinity to glycoproteins bearing sulfated sugars glycoproteins terminating in 4-SO4GalNAc, a fibronectin II (FNII) domain, and eight carbohydrate recognition domains (CRDs) that bind sugars, such as d-mannose and fucose, with high-affinity. Regardless of the continued development of drug delivery technologies, the effective targeting of drugs to macrophages to treat the underlying diseases remains proven [103,121]. Mannosylation is currently the best strategy to develop nanomedicines that target d-mannose receptors, which are highly expressed in cells of the immune system [109,133,134,135]. Based on the growing literature, nanocarrier mannosylation will increase uptake by macrophages to provide clinically relevant concentrations in target tissues or organs.

Moreover, improved uptake is projected to require lower doses of the agents sufficient for therapeutic effects, thus providing reduced toxicity. Researchers have formulated mannosylated polymeric micelles for high efficient delivery of siRNA into macrophages [136]. Bhavin et al. have developed mannosylated PLGA nanoparticles that improve brain bioavailability [133,137]. Moreover, different novel drug delivery approaches in combination with mannosylation for the improvement in selective macrophage uptake, such as polymeric nanoparticle [133,138], polysaccharide-based vaccine [139], liposome [121], niosomes [140], NLC [134], dendrimer [135], solid lipid nanoparticles (SLN) [141], chitosan nanoparticles [62,142], and gelatin nanoparticles [128] have been evaluated.

#### 1.7.2. Passive Targeting

Passive targeting involves accumulating the drug-carrier system at a site due to physicochemical or pharmacological factors [1,42,46]. Nanoparticle accumulation is observed in the liver due to the large fenestrations. The nanocarriers are readily taken up by the monocyte phagocytic system (MPS) cells or reticuloendothelial system (RES) through phagocytosis. RES consists of fixed macrophage cells in organs, mainly the liver (Kupffer cells) and spleen, lung, kidney, bone marrow, circulating monocytes, macrophages, and polymorph nuclear leukocytes cells [143,144]. These RES cells cannot identify the particulate carriers themselves but recognize specialized opsonin proteins deposited on the particle surface, followed by MPS uptake [145].

##### Endocytosis

**Figure 7:**
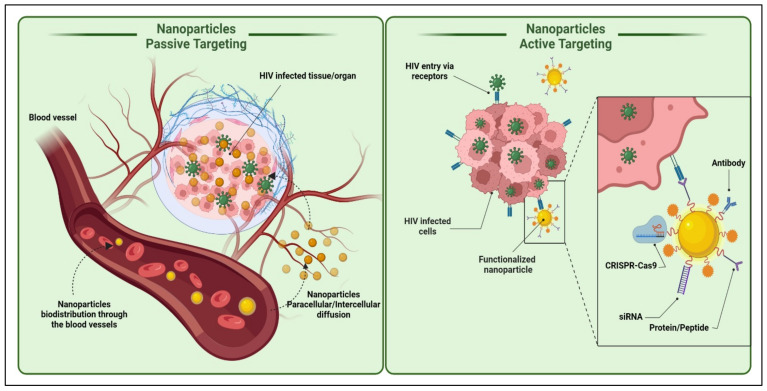
Schematic representation of nanocarrier internalization via various endocytosis mechanisms. (Created with BioRender.com (accessed on 19 June 2022)).

Approaches involving passive targeting can result in the accumulation of higher concentrations of drugs at the target sites. This local gradient difference may allow the drug penetration by passive diffusion. Moreover, trafficking via non-receptor-mediated endocytosis (i.e., macropinocytosis) may enhance cellular drug uptake. Actively targeted drug trafficking can be possible via receptor-mediated endocytosis when the periphery of nanocarriers is tagged with ligand molecules matching the specific cell receptor (Figure 7) [112].

##### Phagocytosis

It is a process by which cells, generally macrophages, engulf solid particles. The first step of phagocytosis is opsonization, in which opsonin (antibody or complement molecules) cover solid particles [146]. Phagocytic cells express Fc and CR1 receptors that bind opsonin molecules and antibodies and complement C3b. Following ingestion, solid particles become trapped in phagocytic vesicles (phagosomes), which fuse with intracellular organelles containing digestive proteins and an acidic internal pH and mature into phagolysosomes that degrade the internalized nano drug delivery system. The nano drug delivery system is then eliminated by exocytosis after degradation or sequestered in residual cells’ bodies if it cannot be digested. Contacts between the nano drug delivery system and macrophages occur via the recognition of opsonin on the nano drug delivery system surface or through interactions with scavenger receptors on macrophages. This fact can target macrophages passively, lymph nodes, and the spleen to treat infections that affect RES (HIV/AIDS) [109,147,148]. Drug or drug carrier nanosystems can be passively targeted by manipulating their physicochemical factors, such as size, shape, surface charge, and surface hydrophobicity [46,130,149].

### 1.8. Factors Impacting the Functionalities of Nanocarrier Targeted Delivery

#### 1.8.1. Particle Size

Particle size affects the bioavailability and circulation time of the nanocarriers [134]. It also decides the mechanism through which it moves in the cell and its localization. The particle size of nanocarriers is suitable for passive targeting of various HIV reservoir sites. Nanoparticles of particle size >200 nm are opsonized, phagocytosed, and taken up by the macrophages of the RES organs, major HIV reservoir sites, while <200 nm escape phagocytosis and localize in remote reservoirs, such as bone marrow, brain, and gonads, in high concentrations [43].

HIV infection is an intracellular infection of macrophages localized primarily in the reticuloendothelial system (RES) organs, including the liver, spleen, lung, lymph node, genitals, lymphocytes, and brain [39]. Treating HIV infection with conventional therapies is unmanageable because of their major prevailing limitations, such as poor efficacy and drug resistance [150]. The drug did not reach the infected site in conventional therapies due to several HIV reservoir barriers [39,96]. Moreover, in the case of nanomedicine, it could passively or actively target these viral reservoir sites and eradicate the HIV infection [42,131,151]. Scientists have been studied the numerous nanomedicine for the improvement of anti-HIV therapies, such as liposome [54,152]; solid lipid nanoparticle [147,153]; polymeric nanoparticle [148,149]; and nanoemulsion [125,154], and nanostructured lipid carriers (NLCs) [143,155].

#### 1.8.2. Particle Shape

More recently, the effect of particle shape on cell uptake and biodistribution has been recognized. In one study, Mitragotri and Champion reported the surprising finding that particle size and particle shape also affect phagocytosis [151,156]. A macrophage internalized ellipse pointed end in a few minutes, while the same ellipse has not internalized via the flat region even over 12 h. Spherical nanoparticles were internalized through any point of attachment because of their symmetry. The asymmetric lipomer of doxycycline hydrochloride and amphotericin B revealed enhanced splenic uptake following intravenous administration [43,157].

#### 1.8.3. Surface Charge

Surface charge and functional groups present on the surface of particulate carrier influence interaction with the cells and further traverse across the negatively charged cell membrane. Positively charged nanoparticles demonstrated higher phagocytic uptake than neutral hydrophilic or negatively charged nanoformulations [158]. For instance, polystyrene nanoparticles with a primary amine group at the surface underwent significantly more phagocytosis than nanoparticles with sulfate, hydroxyl, and carboxyl groups. The extended blood circulation half-life of negatively charged particulate carriers could be due to the reduced adsorption of opsonin [159]. Further, negatively charged nanoparticles could effectively bound to cationic sites on the macrophages at the scavenger receptors, enabling their uptake by RES [160]. The cellular entry of antiretroviral agents that are negatively charged, such as phosphorylated nucleotide analogs and nucleic acids, get faster entry into the cells. The siRNA dendriplexes delivered to human astrocytes decreased the replication of HIV-1 due to higher intracellular concentration [161]. 

#### 1.8.4. Surface Hydrophobicity

The systemic circulation of particulate carriers is strongly influenced by surface hydrophobicity. Particles with hydrophobic surfaces are coated by the complement proteins, albumin, and immunoglobulins and further rapidly cleared by RES (reticuloendothelial system) from the circulation than those with a hydrophilic surface. Surface hydrophobicity thus impacts opsonization, phagocytosis, and the biodistribution of nanoparticles [162,163].

### 1.9. Liposomes-Based Delivery Systems for Anti-HIV Therapeutics

**Figure 8:**
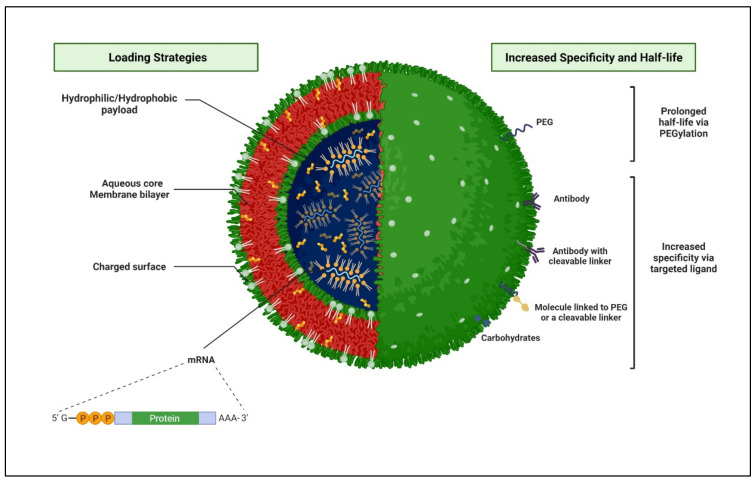
Schematic 3D representation of a structural arrangement of a liposome and various loading strategies, which are leading to enhancing the specificity and half-life of the nanocarrier at the targeted HIV-infected sites. (Created with BioRender.com (accessed on 19 June 2022).

Liposomes are small artificial spherical vesicles constituted by one or more phospholipid bilayers with the polar groups of phospholipids oriented to the inner and outer aqueous phase. Such a structure explains the high propensity of liposomes to be encapsulated with hydrophilic, hydrophobic, and amphiphilic drugs within the inner aqueous compartment, the lipid bilayers, and at their interfaces, respectively (Figure 8).

In this review, NLCs were selected over other nanoparticle constructs because they offer numerous advantages, including controlled release of drug and targeting, improved drug stability, and the capability to incorporate lipophilic and hydrophilic drugs biocompatibility [131,157,164,165]. The latter advantage is that NLCs are made of whichever physiological lipids or lipids are usually used as pharmaceutical excipients [166,167]. The nanocarrier-mediated targeted delivery of anti-retrovirals to HIV reservoirs has revealed great potential [1,46,131,151]. The darunavir-loaded lipid nanoparticle biodistribution study showed higher uptake in the spleen and brain HIV reservoirs [168,169]. Few reports suggest that stavudine-loaded lipid nanocarriers were utilized to deliver effectively to cellular and anatomical HIV reservoirs, increasing therapeutic safety [121,170]. Other studies have reported that nevirapine-loaded SLN and NLCs are used for effective and targeted delivery to HIV reservoirs [167]. In vivo pharmacokinetics assessment of lopinavir-loaded with NLCs in rats has shown a 2.8-fold enhancement in brain uptake [155,167].

Zidovudine (AZT)-loaded docosanol NLCs were confined in the brain in a sustained-release method for an extended period [143]. An AZT-loaded liposome has also shown an improvement in the uptake of lymphoid organs [124]. Significant lymphoid tissue drug localization was found with the indinavir-associated lipid nanoparticles (LNPs) [171]. SLN of efavirenz has shown a substantial amount of efavirenz in the spleen, a major lymphatic organ for better managing HIV [172]. Tenofovir-loaded SLNs improved the cellular uptake of hydrophobic microbicides, which prevent the virus during the infection process and reduce the possible infections [147]. Some of the examples of nanosystems targeting anti-HIV agents to HIV reservoir sites are stated in Table 4.

Moreover, currently, the HIV vaccine is designed using a lipid-based nanoparticle vaccine platform (NVP) that presents HIV-1 viral proteins in a conformational manner for the induction of antigen-specific antibody responses. This type of vaccine can co-deliver protein antigens and adjuvants, which can boost immunogenicity by promoting their distribution to antigen-presenting cells [164]. The current health crisis of coronavirus disease 2019 (COVID-19) highlights the safety of these approaches for generating potent and protective immune responses. In preclinical investigations, nucleoside-modified mRNAs formulated in lipid nanoparticles (mRNA-LNP) have proven to be a strong strategy of vaccination against infectious diseases, and are now being explored in people for SARS-CoV-2 [165].

#### Liposomes-Based Delivery Systems of Ascorbic Acid to Increase the Bioavailability of ARTs

Vitamin C (ascorbic acid) is an essential water-soluble nutrient that functions as a cofactor for numerous enzymatic reactions. Vitamin C also serves as an antioxidant, anti-inflammatory, immunomodulatory, anti-viral, and anti-thrombotic agent and can potentially be used as a therapeutic or prophylactic agent [190]. Preliminary clinical evidence showed that massive doses of ascorbic acid (50–200 g per day) can suppress the symptoms of the HIV disease and can markedly reduce the tendency for secondary infections [191]. Oxidative stress influences viral replication, inflammatory responses, and immune cell proliferation, all of which contribute to the pathogenesis of HIV/AIDS. Hence, vitamin C can be used to reduce the damage caused by oxidative stress. Given the well-defined involvement of several lipids in the physiology of phagocytosis, the use of bioactive lipid nanoparticles to alter the phagosome maturation process has been proposed as a way to boost the efficacy of innate immunity mechanisms [192].

In this context, many experimental findings suggested that a liposome-based delivery system of ascorbic acid may be potentially beneficial to reduce oxidative stress and prevent several HIV chronic conditions and immune system activation. This strategy might help the oral administration of the ARTs [193]. However, more research and trials are needed to determine the effects of vitamin C on HIV disease progression and prognosis.

### 1.10. Nanotechnological Advantages for Effective Anti-HIV Therapy

Nanotechnology-based nanosize drug-loaded carrier design presents manifold advantages, including targeting different anatomical and cellular viral reservoirs, thereby completely eradicating the virus from the reservoir sites. Targeted drug delivery at the site of action improves the drug’s efficacy and reduces the off-target effect. Nanocarriers deliver drugs in a controlled manner, increasing residence time at the target sites, increasing bioavailability, and improving the quality of life of HIV patients [125,194].

More importantly, a salient feature of nanotechnology that can be exploited for anti-HIV therapy is altered *in vivo* biodistribution [1,195,196,197]. Although conventional drug distribution in the body is based on drug physicochemical properties, the drug properties are overshadowed to achieve carrier-mediated distribution when loaded in nanocarriers [198,199]. The physicochemical properties of the nanocarriers then become the rate-determining factor for the distribution in the body [200,201,202,203,204,205]. Therefore, altering the same could provide a promising approach to targeting HIV reservoir sites for effective anti-HIV therapy. Targeted drug delivery could be achieved via passive or active targeting [57,206,207].

## 2. Future Prospective

The major challenge in HIV infection treatment is targeting remote reservoirs, such as the brain, bone marrow, testicles, and cellular sites. Newer drug delivery options should be considered to treat HIV infection to effectively target these sites. Moreover, PBPK modeling is recommended to design the long-acting HIV formulations for the rapid development of long-acting technologies. There is also a dire need to focus on emerging technologies, such as microneedles, polymeric implants, nanorobots, HIV vaccines, and any such synergistic combination of technologies, that could be helpful to target HIV reservoir sites and provide long-acting effects that may lead to nearly curing HIV infection. There is also a need to explore the underlying mechanisms for HIV reactivation and drug delivery strategies that could effectively target the viruses. Adjuvant therapies that could simultaneously manage the eradication of HIV reservoir sites while minimizing the cellular oxidative stress to improve could also be explored for a more patient-compliant HIV infection therapy.

## 3. Conclusions

Several drug delivery strategies have been developed and proposed to minimize and eradicate HIV from remote reservoirs. However, there are still obstacles, such as low efficacy and nontargeting effect, and dose-related toxicities. Nanopharmaceuticals offer excellent treatment options for HIV infections by improving the drug potency/efficacy, lowering the dose-related toxicities, and providing active targeting options to the remote HIV reservoirs, leading to the near-total eradication of the virus. In addition, nanopharmaceuticals offer advantages over conventional drug delivery, such as an encapsulation of the drug in nanocarriers, despite its physiochemical properties, providing long-acting treatment options and reducing the dosing because of selective targeting and improvements to the bioavailability of the hydrophobic drugs and adherence of the patients. Moreover, it reduces the adverse effects of the drug delivery system because of its selectivity and specificity. These technologies could be formulated in combinations, providing the synergistic effect for improved therapy.

There are still several obstacles to translate the nanocarrier systems easily from the lab to the clinics. Thus, there is a dire need to develop the translational nanocarrier, which could be helpful in HIV infection treatment. The issue of long-term toxicity and in vivo stability should be prioritized in the design of nanoparticles. Moreover, interdisciplinary associations on nanomedicine development, characterization and evaluation, and suitable preclinical and in vitro models offer the best way forward for clinical translation of nanomedicines for HIV treatment.

## Figures and Tables

**Table 1:** Available CDC-approved ART drugs used in HAART therapy.

Class of Drug	Drug
Nucleoside Reverse Transcriptase Inhibitors (NRTIs)	Abacavir, Didanosine, Lamivudine, Stavudine, Zalcitabine, Zidovudine
Nucleoside Reverse Transcriptase Inhibitor (NRTIs)	Delavirdine, Efavirenz, Nevirapine
Nucleotide Reverse Transcriptase Inhibitors (NtRTIs)	Tenofovir diisoproxil fumarate
Protease Inhibitors (PIs)	Amprenavir, Indinavir, Lopinavir, Ritonavir, Nelfinavir, Saquinavir
Fusion Inhibitors (FIs)	Enfuvirtide
Co-Receptor Inhibitors (CRIs)	Maraviroc

**Table 2:** Macrophage receptors and their specific ligands.

Receptor	Ligands
d-Mannose	d-Mannose, fucose, N-acetyl glucose-mine, glucose, collagen, mannan, mannosyl lipoarabinomannan [103,104]
Folate	Folic acid [105,106]
Tuftsin	Tuftsin [107]
Scavenger	Modified LDL, lipopolysaccharides, lipoteichoic acid [108]
Transferrin	Transferrin [109]
Fc	Monoclonal Antibody [110]
Fibronectin	Fibronectin, laminin, serum amyloid P [117]
Toll-like receptor	LPS, lipoproteins, lipopeptides, and lipoarabinomannan [118]
Complement Receptors (CR3 and CR4)	C3b, iC3b, C3 [119]

**Table 3:** Drug delivery systems are exploited for targeted delivery of anti-HIV1 drugs to reservoir sites.

Nanocarrier and Targeting Ligand	Drug	Targeting Sites
**Liposomes**		
β-d-1-thiomannopyr-anoside	Indinavir	Liver, spleen, and lungs [120]
d-mannose	Stavudine	Maintained significant levels in the liver, spleen, and lungs and overcame the development of anemia and leukocytopenia [121]
Galactose	Stavudine	Prolonged residence in liver and spleen [122]
Galactose	Azidothymidine palmitate	Liver [123]
Galactose	Azidothymidine	Prolonged residence in the body [122]
d-mannose	Zidovudine	Lymph nodes and liver [124]
Antibodies against human and murine HLA-DR and CD4 antigen	Indinavir	Lymph nodes, liver, spleen, and plasma [101]
**Nanoparticles**		
Transferrin	Azidothymidine	Brain [125,126]
Mannan	Didanosine	Spleen, lymph nodes, and brain [127]
d-mannose	Didanosine	Lung, liver, and lymph nodes [128]
Trans-Activating Transcriptor (TAT) peptide	Ritonavir	Brain [129]
**SLN**		
Transferrin	Saquinavir	Brain microvascular endothelial cells [130]
Bovine serum albumin	Stavudine	Liver, spleen, brain [131,132]
Dextran	Stavudine	Liver, spleen, brain [132]

**Table 4:** Particle size-based nanocarriers for passive targeting nanocarriers.

Drug	Particle Size	Targeting Sites
**Liposomes**		
Stavudine	120 ± 1.52 nm	Liver, spleen, and lungs [121]
Deoxycytidine	300 nm	Reduced proviral DNA in mononuclear phagocyte system cells of spleen and bone marrow [173]
Foscarnet		Enhanced the drug localization in RES organs [144]
2′,3′-dideoxyinosine	112 nm and 83 nm	Lymph nodes, liver, spleen [145]
Zidovudine	130–160 nm	Lymph nodes, liver, spleen, plasma [174]
Zidovudine	90 nm	Organs of RES and brain [175]
Zidovudine	120 ± 10 nm	Spleen and lymph nodes [124]
**Solid Lipid nanoparticles**		
Lopinavir-Ritonavir	223 nm	Liver, spleen, mesenteric lymph nodes, and plasma [176,177]
Zidovudine	181 ± 26 nm	Liver [123]
Lopinavir	230 nm	Plasma and cerebrospinal fluid [16]
Zidovudine	600–630 nm	Brain and liver [178]
Stavudine	75 nm	Liver, spleen and lung [132]
Stavudine	175 ± 6 nm	Liver, spleen, lungs, bone marrow, lymph nodes, and brain [179]
Efavirenz	124.5–362 nm	Plasma [180]
Atazanavir	167 nm	Enhanced accumulation in human brain microvessel endothelial cell line [181]
**Polymeric nanoparticles**		
Zidovudine	230 ± 20 nm	RES organs and plasma [182]
Indinavir	1.6 um	Lung, liver, spleen, and bone marrow-derived macrophages [183]
Zidovudine	56 to 93 nm	Brain, liver, and spleen [184]
Atazanavir	268 nm	Liver and spleen [185]
Ritonavir, lopinavir and efavirenz	331.2 ± 77.2 nm	Serum, brain, liver spleen, testes [176]
Efavirenz, lopinavir and ritonavir	138.3 ± 55.4 nm	Enhanced cellular uptake and anti-HIV activity in H9 T cells [138]
Nevirapine	450–550 nm	Brain, liver, and spleen [186]
Indinavir	210 nm	Brain [187]
Rilpivirine	200 nm	Sustained release in plasma [188]
**Dendrimer**		
Lamivudine	≈ 200 nm	Significantly enhanced uptake and anti-HIV activity [189]

## Data Availability

Data are contained within the article.
